# Supplementary figures and images for: Automated evaluation of quaternary structures from protein crystals
Source: PLoS Comput Biol. 2018 Apr 30;14(4):e1006104. doi: 10.1371/journal.pcbi.1006104 (PMC5945228; doi:10.1371/journal.pcbi.1006104)

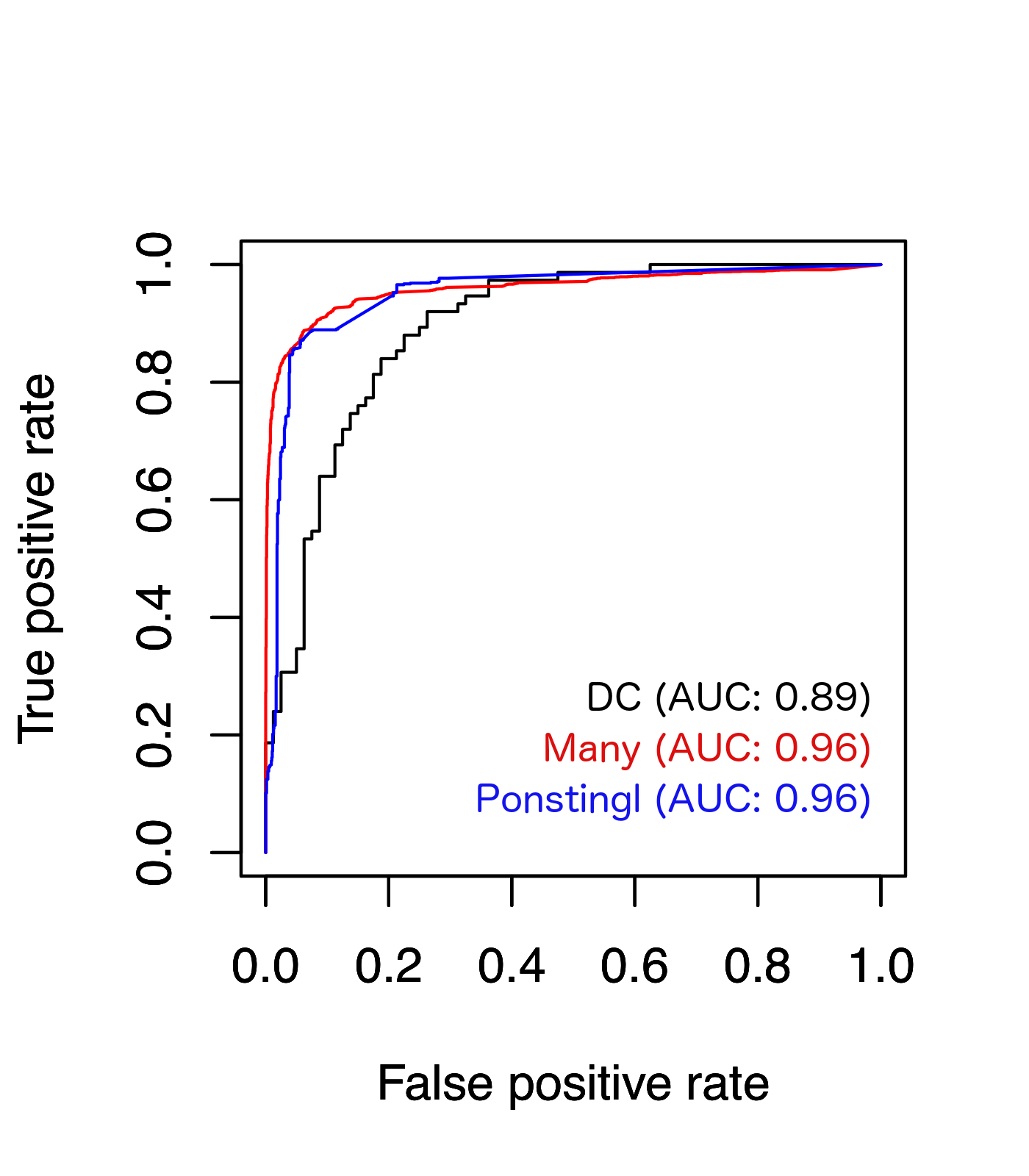

Supplement: S1 Fig — The curve is directly comparable to those appeared in Baskaran et al. 2014 BMC Structural Biology. (TIF) [file pcbi.1006104.s001.tif]
